# The advanced machine learner XGBoost did not reduce prehospital trauma mistriage compared with logistic regression: a simulation study

**DOI:** 10.1186/s12911-021-01558-y

**Published:** 2021-06-21

**Authors:** Anna Larsson, Johanna Berg, Mikael Gellerfors, Martin Gerdin Wärnberg

**Affiliations:** 1 Emergency Department, Södersjukhuset, Sjukhusbacken 10, 11883 Stockholm, Sweden; 2 Department of Emergency Medicine, Skåne University Hospital Malmö, Inga Marie Nilssons gata 47, 21421 Malmö, Sweden; 3 Department of Global Public Health, Karolinska Institutet, 171 77 Solna, Sweden; 4 Department of Physiology and Pharmacology, Karolinska Institutet, 171 77 Solna, Sweden; 5 Function Perioperative Medicine and Intensive Care, Karolinska University Hospital, Solna, Stockholm, Sweden; 6 Swedish Air Ambulance (SLA), Mora, Sweden; 7 Rapid Response Cars, Stockholm, Sweden

**Keywords:** Trauma, Prehospital triage, Undertriage, Overtriage, Clinical prediction model, Machine learning

## Abstract

**Background:**

Accurate prehospital trauma triage is crucial for identifying critically injured patients and determining the level of care. In the prehospital setting, time and data are often scarce, limiting the complexity of triage models. The aim of this study was to assess whether, compared with logistic regression, the advanced machine learner XGBoost *(eXtreme Gradient Boosting)* is associated with reduced prehospital trauma mistriage.

**Methods:**

We conducted a simulation study based on data from the US National Trauma Data Bank (NTDB) and the Swedish Trauma Registry (SweTrau). We used categorized systolic blood pressure, respiratory rate, Glasgow Coma Scale and age as our predictors. The outcome was the difference in under- and overtriage rates between the models for different training dataset sizes.

**Results:**

We used data from 813,567 patients in the NTDB and 30,577 patients in SweTrau. In SweTrau, the smallest training set of 10 events per free parameter was sufficient for model development. XGBoost achieved undertriage rates in the range of 0.314–0.324 with corresponding overtriage rates of 0.319–0.322. Logistic regression achieved undertriage rates ranging from 0.312 to 0.321 with associated overtriage rates ranging from 0.321 to 0.323. In NTDB, XGBoost required the largest training set size of 1000 events per free parameter to achieve robust results, whereas logistic regression achieved stable performance from a training set size of 25 events per free parameter. For the training set size of 1000 events per free parameter, XGBoost obtained an undertriage rate of 0.406 with an overtriage of 0.463. For logistic regression, the corresponding undertriage was 0.395 with an overtriage of 0.468.

**Conclusion:**

The under- and overtriage rates associated with the advanced machine learner XGBoost were similar to the rates associated with logistic regression regardless of sample size, but XGBoost required larger training sets to obtain robust results. We do not recommend using XGBoost over logistic regression in this context when predictors are few and categorical.

**Supplementary Information:**

The online version contains supplementary material available at 10.1186/s12911-021-01558-y.

## Background

Accurate prehospital trauma triage is crucial for identifying critically injured patients and determining where to transport these patients [1]. In this context, undertriage (false negative) is when a patient requiring specialized trauma care is transferred to a lower-level trauma centre and is associated with higher mortality rates [1, 2]. Conversely, overtriage (false positive) occurs when a patient not in need of specialized trauma care is transferred to a higher-level trauma centre, resulting in extra costs and overutilization of resources [3].

The American College of Surgeons Committee on Trauma (ACS-COT) guidelines state that trauma systems must aim to attain a maximum of 5% undertriage and keep overtriage below 35% [4]. Extensive research has focused on the development of prediction models to assist emergency medical service (EMS) personnel in the early clinical decision making and triage process [5]. However, most existing trauma triage protocols perform poorly, leading to high rates of mistriage [6–8].

Prehospital trauma triage is particularly challenging due to a lack of time and patient information [9]. Previous research shows that prediction models incorporating several categories of predictors (physiological, demographic, anatomical, etc.) generally perform better than simpler models [5]. However, in the prehospital setting, patient information is often scarce, and health care providers need to prioritize stabilization and rapid transport over a thorough medical history [10, 11].

Extensive research has focused on how machine learning can improve predictions and diagnosis in medicine [12, 13]. Studies show that, given comprehensive input parameters, machine learning may outperform classical statistical methods in predicting the need for critical care and hospitalization in the emergency department and prehospital setting [14–17]. Other research indicates that logistic regression may perform as well as machine learners when used to develop clinical prediction models [18–20].

Little is known about how much data are needed and how complex the data must be to obtain accurate predictions with machine learners. Predictive accuracy is adversely affected when a model is transferred from one setting to another [21]; therefore, it is desirable to use models that can be developed from available local data. We also know that health care professionals are not willing to use complex systems disrupting their workflow and requiring time away from the patient [22].

We wanted to study whether an advanced machine learner could bring any added value to prehospital trauma triage, given limited input data complexity. Our aim was therefore to assess whether, compared with logistic regression, which is a classical modelling technique commonly used in this context, the advanced machine learner XGBoost *(eXtreme Gradient Boosting)* is associated with reduced prehospital trauma mistriage. To estimate how much data were needed for model development, we assessed the performance of the learners for different training data set sizes. We chose the machine learner (XGBoost) because it has recently been dominating applied machine learning and Kaggle competitions [23, 24].

## Methods

### Study design

We conducted a simulation study based on data from the US National Trauma Bank (NTDB) and the Swedish trauma registry (SweTrau). The NTDB cohort included a total of 813,567 patients enrolled in the NTDB in 2014. The SweTrau cohort included a total of 30,577 patients registered in the Swedish trauma registry between 2011 and 2016.

### Variables

#### Outcome

The outcome of the study was the pairwise difference in over- and undertriage rates between the models. We used an ISS > 15 as the gold standard to define trauma severity as major trauma. Patients with an ISS ≤ 15 were considered to have minor trauma [4]. We defined the overtriage rate as the false-positive rate and the undertriage rate as the false-negative rate.

- Overtriage rate (false-positive rate) = Number of patients with an ISS ≤ 15 classified as major trauma/total number of minor trauma patients (ISS ≤ 15)
- Undertriage rate (false-negative rate) = Number of patients with an ISS > 15 classified as minor trauma/total number of major trauma patients (ISS > 15)

By definition: Specificity + False-positive rate = 1, Sensitivity + False-negative rate = 1.

#### Predictors

The predictors used to build our models were systolic blood pressure, respiratory rate, Glasgow Coma Scale (GCS) and age. Our rationale was that these parameters are known to be predictive of mortality after trauma and are easily collected by EMS personnel in the prehospital setting. We used the first recorded vital parameters at the scene of the injury. The vital parameters were categorized according to the Revised Trauma Score (RTS) [25]. Our rationale for categorizing these variables was that in severe trauma, it can be hard to obtain an exact count of the respiratory rate, GCS or an accurate measure of a very low blood pressure. We included age as a significant risk factor for mortality in trauma, with a significant increase from 57 years of age [26]. Additionally, due to the loss of regulatory and adaptive mechanisms, vital signs respond differently to stressors in elderly individuals [27].

### Data

SweTrau is a nationally encompassing registry in which 92% of Swedish hospitals record trauma cases. The inclusion criteria were as follows: traumatic events with subsequent activation of the hospital trauma protocol, admitted patients with an NISS > 15 and patients transferred to the hospital within 7 days of traumatic events with an NISS > 15. SweTrau excludes patients if the only injury is chronic subdural haematoma or if the hospital trauma protocol is activated without traumatic events [28].

The US National Trauma Data Bank (NTDB) is the largest aggregation of U.S. trauma registry data ever assembled. Currently, the NTDB contains detailed data on over six million cases from over 900 registered U.S. trauma centres [29]. The NTDB includes any patient with at least one injury diagnosis code (ICD-9CM 800–959.9), excluding late effects of injury, superficial injuries, and foreign bodies [30]. In addition, patients must be admitted as trauma patients, transferred from another institution or have died as a result of their injury [30].

#### Eligibility criteria

The inclusion criteria of the present study were age above 15. Observations with unrealistic recordings of SBP > 300 and RR > 67 were excluded. Observations with systolic blood pressure of 0 were excluded due to uncertain underlying reasons ranging from unsuccessful recording to cardiac arrest. Observations with missing values were excluded.

#### Study size

As real-world data are often scarce, we wanted to estimate how much data are actually needed to develop a reliable model. We therefore performed the study for different training set sizes. The required study size for each phase of model development using logistic regression is fairly well established, i.e., for derivation at least 10 events per free parameter in the potentially most complex model and for validation at least 100 events, assuming that events are more common than non-events. No study size guidelines exist for the ML algorithm XGBoost. We therefore trained the models in training sets of sizes 10, 25, 100 and 1000 events per free parameter.

### Model development

We used R for the statistical analyses and development of the models. The following steps were followed for each of the cohorts and training set size. First, a training set was created using a simple random draw from the complete cohort. The size of the training set was NX/P, where N is the number of free parameters, X is the number of events per free parameter and P is the proportion of events in the cohort. Each of the models was trained in this training set. Second, the validation and test sets were created using simple random sampling from the complete cohort. The size of the validation and test sets was 200/P, where P is the proportion of events in the cohort. We used the validation set to define the cut-off probability for major and minor trauma. The rationale for using the validation set and not the training set for this was to improve the generalization properties of the models.

The optimal cut-off was identified by performing a gridsearch on the output probabilities of the validation set, evaluating the over- and undertriage rates for every cut-off probability. The gridsearch was performed from 0 to 1 in steps of 0.001. This cut-off was set to aim at an undertriage rate below or equal to 5% with as low overtriage as possible to be in accordance with ACS-COT guidelines [4]. In the absence of undertriage below 5%, we set the cut-off to obtain as low undertriage as possible with an upper limit for overtriage below 50%. This cut-off was then applied to the output probabilities from the test set to compare the performance of the final models.

The exact performance of a model depends on the subsets of data used for development, validation and testing. We therefore repeated the above process 1000 times for every size of training set and cohort. The results are presented as the median and 2.5% and 97.5% percentiles across bootstrap samples.

#### Logistic regression

A logistic regression model was created using bootstrapping to limit overfitting and optimism according to current guidelines [31]. The following steps were followed. First, the model was fit using all data in the training set, creating an original model M_0_. Then, a bootstrap sample of size N was drawn with replacement from the training set, and the model was fit using this bootstrap sample, resulting in a bootstrap model M_bs,i._ M_bs,i_ was then used to predict the outcomes in the training set. The resulting linear predictor y was regressed on the outcomes Y using a logistic model. The coefficients of the linear predictor, denoted b_i_, where i is the number of bootstrap samples, were stored. This procedure was repeated 1000 times, producing v = b_1_, b_2_, b_3_,..., b_1__000_. $\bar{\text{v}}$ was finally used to shrink the coefficients of the original model as $\bar{\text{v}} \times {\text {M}}_0$.

#### Extreme gradient boosting (XGBoost)

XGBoost is an implementation of gradient boosted decision trees [23]. Gradient boosting is an approach where new models are created that predict the residuals or errors of prior models and then combined to make the final prediction.

The XGBoost model was developed using the R package “xgboost”. The hyperparameters *nrounds*, *eta*, *max_depth* and *lambda* were tuned using the r package “MLR” [32]. For the other hyperparameters, we used default values. The hyperparameters were tuned using MLR functions following a well-defined procedure. We defined the searchspace for the parameters to be tuned and performed a random search using fivefold cross-validation. This approach implied that our training and validation data were combined and then split into 5 equally sized parts. The model was trained on four of the five parts and evaluated on the 5th. This process was repeated until each of the parts had been used as the validation set. The hyperparameters giving the best performance were selected for the training of the final XGBoost model. The final XGBoost model was trained using only the training data set.

### Performance measures

Model performance was assessed in terms of over- and undertriage rates as defined in Sect. 2.2.1. Additionally, we calculated the sensitivity, specificity, area under the curve (AUC), calibration slope and calibration intercept for the models. The over- and undertriage rates are presented in detail in the results section. AUC and calibration properties are mentioned in the results section and presented in detail as supplementary material. As sensitivity and specificity can be directly calculated from the over- and undertriage rates, we have chosen to only present these measures as supplementary material.

## Results

The study was conducted in the NTDB and SweTrau cohorts in parallel (Table 1). The NTDB cohort included a total of 813,567 patients enrolled in the National Trauma Data Bank (NTDB) during 2014. The SweTrau cohort included a total of 30,577 patients registered in the Swedish trauma registry between 2011 and 2016. After excluding observations with missing recordings and applying the inclusion criteria, we were left with 368,810 observations in the NTDB cohort and 16,547 observations in the SweTrau cohort. The proportion of major trauma events in the NTDB was larger than that in SweTrau, probably because it only included patients admitted to the hospital.

**Table 1:** Characteristics of study data and sizes of data sets

Characteristics	NTDB	SweTrau
Total number of observations	813,567	30,577
Number of missing observations	422,416	10,411
Number of included observations	368,810	16,547
Proportion major trauma	0.21	0.12
Proportion female	0.38	0.35
Age (median-IQR)	51 [30, 69]	41 [25 59]

GCS Category	NTDB proportion of observations	SweTrau proportion of observations
13–15	0.9098	0.9173
9–12	0.0384	0.0401
6–8	0.0175	0.018
4–5	0.0061	0.009
3	0.0283	0.0156

RR Category	NTDB proportion of observations	SweTrau proportion of observations
30–67	0.0191	0.0543
10–29	0.9677	0.9407
6–9	0.0063	0.0036
0–5	0.0021	0.0011

SBP Category	NTDB proportion of observations	SweTrau proportion of observations
90–300	0.9707	0.9798
76–89	0.0192	0.0126
50–75	0.0091	0.0068
1–49	0.0011	0.0007

Size training sets (events per free parameter)	NTDB	SweTrau
10	714	1250
25	1786	3125
100	7143	12,500
1000	71,429	Missing

Size validation and test sets	NTDB	SweTrau
(200/proportion events)	952	1667

NTDB, National Trauma Data Bank; SweTrau, Swedish Trauma Registry; GCS, Glasgow Coma Scale; RR, Respiratory Rate; SBP, Systolic Blood Pressure

### Performance of models

In SweTrau, the smallest training set of 10 events per free parameter was sufficient to achieve robust results (Table 2). XGBoost obtained undertriage rates in the range of 0.314–0.324 with corresponding overtriage rates of 0.322–0.319. In SweTrau logistic regression achieved undertriage rates ranging from 0.312 to 0.321, with overtriage rates ranging from 0.323 to 0.321. The area under the curve (AUC), calibration slope and calibration intercept were calculated and are presented in Additional file 1: Tables S1 and S2. In SweTrau, XGBoost obtained a maximal AUC of 0.725 with a corresponding calibration slope of 1.056 and calibration intercept of 0.009. The best discrimination and calibration properties for logistic regression were an AUC of 0.725 with a calibration slope of 1.01 and calibration intercept of -0.017.

**Table 2:** Under- and overtriage rates of logistic regression and XGBoost (median and 2.5 and 97.5 percentiles (calculation on 1000 runs))

Events per free parameter	Data set	Undertriage logistic regression	Overtriage logistic regression	Undertriage XGBoost	Overtriage XGBoost
10	SweTrau	0.321 [0.259, 0.389]	0.321 [0.299, 0.344]	0.324 [0.258, 0.683]	0.319 [0.057, 0.344]
10	NTDB	0.429 [0.338, 0.79]	0.453 [0.052, 0.501]	0.701 [0.35, 0.808]	0.08 [0.039, 0.494]
25	SweTrau	0.314 [0.257, 0.379]	0.322 [0.3, 0.346]	0.316 [0.258, 0.61]	0.321 [0.09, 0.345]
25	NTDB	0.405 [0.332, 0.771]	0.46 [0.06, 0.499]	0.436 [0.345, 0.792]	0.444 [0.045, 0.498]
100	SweTrau	0.312 [0.254, 0.373]	0.323 [0.301, 0.345]	0.314 [0.255, 0.4]	0.322 [0.291, 0.345]
100	NTDB	0.394 [0.324, 0.735]	0.466 [0.072, 0.503]	0.409 [0.327, 0.79]	0.459 [0.048, 0.497]
1000	NTDB	0.395 [0.327, 0.72]	0.468 [0.078, 0.507]	0.406 [0.328, 0.777]	0.463 [0.05, 0.504]

NTDB, National Trauma Data Bank; SweTrau, Swedish Trauma Registry

In NTDB, XGBoost required the largest training set size of 1000 events per free parameter to achieve robust results, whereas logistic regression achieved stable results from a training set size of 25 events per free parameter. For the training set size of 1000 events per free parameter, XGBoost achieved an undertriage rate of 0.406 with an associated overtriage rate of 0.463. The corresponding AUC was 0.611 with a calibration slope of 1.097 and calibration intercept of − 0.021. For logistic regression, the corresponding undertriage was 0.395 with overtriage of 0.468 with an AUC of 0.614, calibration slope of 0.995 and calibration intercept of − 0.015.

**Fig. 1:**
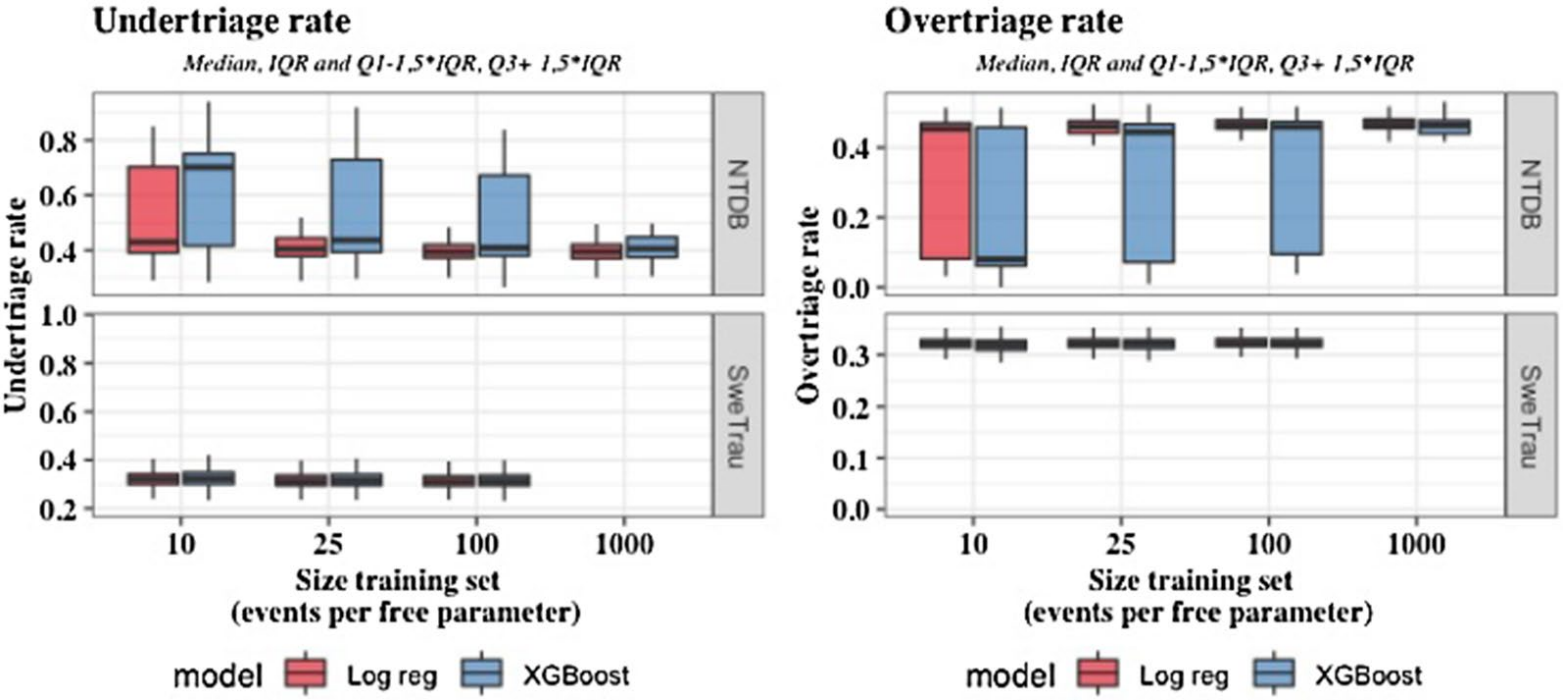
Under- and overtriage rates (median, IQR (Q1-Q3), Q1-1,5IQR & Q3 + 1,5IQR) for logistic regression and XGBoost in the SweTrau and NTDB cohorts. NTDB, National Trauma Data Bank; SweTrau, Swedish Trauma Registry

Overall, as shown in Fig. 1 and Table 3, the predictive performance was better in SweTrau than in NTDB.

**Table 3:** Median and 2.5 and 97.5 percentiles of difference in under- and overtriage rates between learners (calculation on 1000 runs)

Events per free parameter	Data set	Difference in undertriage LogReg-XGBoost	Difference in overtriage LogReg-XGBoost
10	SweTrau	0 [− 0.354, 0.025]	0 [− 0.005, 0.265]
10	NTDB	− 0.005 [− 0.398, 0.035]	0.004 [− 0.017, 0.433]
25	SweTrau	0 [− 0.301, 0.015]	0 [− 0.003, 0.238]
25	NTDB	− 0.005 [− 0.39, 0.023]	0.003 [− 0.015, 0.427]
100	SweTrau	0 [− 0.025, 0.005]	0 [− 0.001, 0.008]
100	NTDB	0 [− 0.396, 0.005]	0 [− 0.003, 0.427]
1000	NTDB	0 [− 0.386, 0]	0 [− 0.001, 0.422]

NTDB, National Trauma Data Bank; SweTrau, Swedish Trauma Registry

### Comparison of model performance

**Fig. 2:**
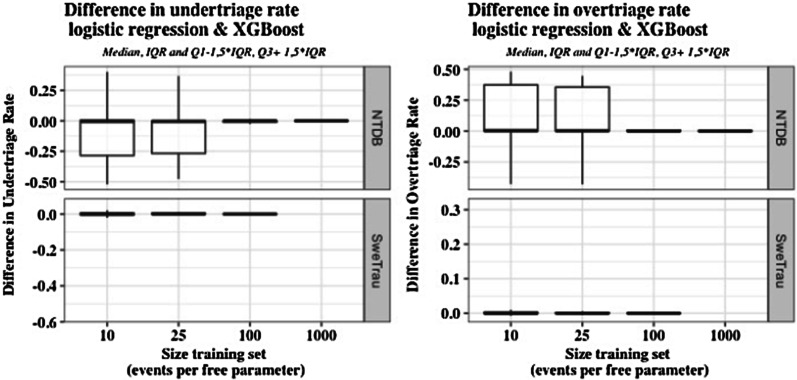
Differences in under- and overtriage rates between learners (median, IQR (Q1-Q3), Q1-1,5*IQR & Q3* + *1,5*IQR) in the SweTrau and NTDB cohorts. NTDB, National Trauma Data Bank; SweTrau, Swedish Trauma Registry

The performance of XGBoost and logistic regression was compared in each of the 1000 runs, i.e., the models were compared when they had been developed on the same set of training data and evaluated on the same set of test data. Table 3 and Fig. 2 show the difference in under- and overtriage rates between the models for these 1000 repetitions (median and 2.5, 97.5 percentiles).

In SweTrau, there were only minimal differences in performance between the models. As seen from Table 3, the median of the difference in performance in SweTrau was 0 for all training set sizes. In NTDB, logistic regression achieved stable results from a training set size of 25 events per free parameter, whereas XGBoost required the largest training set size of 1000 events per free parameter to achieve stable results. Consequently, there were differences in performance for the smaller training sets with a clear advantage for logistic regression. For the largest training data set of 1000 events per free parameter, there were only minimal differences in performance between the models.

## Discussion

The aim of this study was to determine whether, compared to logistic regression, the advanced machine learner XGBoost could bring any added value to prehospital trauma triage. We found that differences in mistriage rates were generally very small, using few categorized predictors and regardless of sample size, but that XGBoost required more data to provide robust estimates.

The time-sensitive nature of trauma makes it difficult for EMS personnel to gather a full medical history and perform a meticulous exam. The prehospital decision to consider the patient seriously injured followed by field triage to a trauma unit is often based on limited information. It is therefore advantageous to develop reliable models that depend only on easily accessible data such as vital parameters and age.

Both models achieved the best possible overtriage of 32% with the best possible undertriage of 31%. Thus, in SweTrau, the overtriage was in line with the ACS-COT recommendation of maximum 35% overtriage, but the undertriage of 31% was far from the ACS-COT recommendation of maximum 5%. The performance of the models was better in SweTrau than in NTDB. This is likely a reflection of a different distribution of the input vital parameters in relation to the proportion of major trauma output in the two cohorts. Despite the higher proportion of events in the NTDB cohort, the categorical distribution of vital parameters was similar in the two cohorts. This naturally implies different conditions for the modelling task. This also enlightens the complexity of modelling, showing how the performance of the same learner may vary from one setting to another.

In SweTrau, both models produced robust results for all training set sizes, and there were only minimal differences in performance. In NTDB, however, logistic regression produced reliable results from the training set of 25 events per free parameter, whereas XGBoost required the largest data set of 1000 events per parameter to stabilize. Thus, the results indicate that XGBoost may require larger and more comprehensive training data than logistic regression to produce robust results. In addition, as opposed to XGBoost, logistic regression has the advantage of being more transparent.

Previous studies have shown variable results when comparing logistic regression to machine learning for clinical prediction models. Overall, it seems that for smaller studies with a limited number of predictors, logistic regression performs as well as more advanced machine learners [19, 20], whereas for larger studies with many predictors, more advanced machine learners may have an advantage [14–17]. A recent review including 71 studies with a median of 19 predictors and 8 events per predictor showed no benefits of machine learning over logistic regression [18], but the included studies did not investigate which factors influenced performance. Further studies are needed to establish determinants of the performance of different algorithms.

Our results show that static vital parameters and age are not sufficient as input parameters to achieve mistriage rates in line with the ACS-COT recommendations. To improve predictive accuracy, vital parameters could be encoded differently, for instance, using the New Trauma Score (NTS) [33] and shock index [34]. Additional predictors, such as trauma type, mechanism of injury or anatomical location of injury, could also be introduced. However, increased model complexity and creation of comprehensive data registries need to be balanced against the clinical reality of EMS personnel in the prehospital setting.

Identifying the optimal prediction model for trauma team activation may enable the development of prehospital and early inhospital decision-making tools to assist EMS personnel in field triage. This tool may also reduce the prehospital provider interindividual variation in the assessment of injury severity, creating a more solid trauma team activation field triage system.

For example, a recent study showed that only 50% of seriously injured patients in Norway are treated by anaesthesiology-manned prehospital critical care teams [35]. Prehospital undertriage may lead to negative patient outcomes. Analysing selected dispatch data with previously described learners may assist dispatch centres in correctly dispatching a high tier unit to a trauma scene with severely injured patients.

### Limitations

Neither SweTrau nor NTDB are perfect populations for developing a trauma triage tool. The ideal population would have been all trauma cases reported to the EMS. Additionally, we excluded data with missing observations. Thus, there were possible sources of selection bias; however, as our objective was to compare two learners, this bias is unlikely to have any major impact on our findings. If the goal was to develop a new model, we would recommend a different approach for handling missing observations, such as multiple imputation.

Encoding of data as categorical variables implies loss of information and could introduce bias. For instance, it is unlikely that the prognosis of an individual would change drastically on the day of the 57th birthday. However, categorical encoding is the norm in published models for prehospital trauma triage, as it can be difficult to count an exact respiratory rate or GCS in a stressful situation.

There are other machine learners for binary classification that we could have assessed, but we used XGBoost because of its current popularity and applicability. Our results cannot be generalized to other machine learners and are limited to XGBoost, as it is implemented in the R package with the same name. It is possible that more extensive hyperparameter tuning, including data re-balancing techniques, would have improved the performance of both learners, but the use of the R package MLR allowed us to replicate the analysis process in all bootstrap samples. This would not have been possible if we had tuned hyperparameters manually.

## Conclusion

The results showed that the advanced machine learner XGBoost did not bring any added value over logistic regression for prehospital trauma triage. In contrast, we observed that this advanced machine learner required larger data sets to produce reliable results. Thus, when predictors are few and categorical, logistic regression may be preferable to this more advanced machine learner.

## Supplementary Information

**Additional file 1.** Additional model performance results.

## Data Availability

The data that support the findings of this study are available from the US American College of Surgeons and the Swedish Trauma Registry, but restrictions apply to the availability of these data, which were used under licence for the current study and so are not publicly available. To access the data from this study, please request the appropriate datasets from the US American College of Surgeons (https://www.facs.org/quality-programs/trauma/tqp/center-programs/ntdb) and the Swedish Trauma Registry (http://rcsyd.se/swetrau/om-swetrau/about-swetrau-in-english/swetrau-the-swedish-trauma-registry) or contact Martin Gerdin Wärnberg (martin.gerdin@ki.se) for more information.

## Abbreviations

ACS COT: The American College of Surgeons Committee on Trauma
AUC: Area Under the Curve
EMS: Emergency Medical Service (Ambulance services, paramedic services)
GCS: Glasgow Coma Scale
NTDB: National Trauma Data Bank
RTS: Revised Trauma Score
SweTrau: Swedish Trauma Registry
XGBoost: Extreme Gradient Boosting
