# Supplementary material for: The advanced machine learner XGBoost did not reduce prehospital trauma mistriage compared with logistic regression: a simulation study
Source: BMC Med Inform Decis Mak. 2021 Jun 21;21:192. doi: 10.1186/s12911-021-01558-y (PMC8215793; doi:10.1186/s12911-021-01558-y)
Supplement: Supplementary file 1 — Additional file 1. Additional model performance results. [file 12911_2021_1558_MOESM1_ESM.docx]

**Supplementary Material**

**Table S1. Sensitivity and specificity**

| **Events per free parameter** | **Data set** | **Sensitivity LogReg** | **Specificity LogReg** | **Sensitivity XGBoost** | **Specificity XGBoost** |
| --- | --- | --- | --- | --- | --- |
| 10 | SweTrau | 0.679 [ 0.611, 0.741 ] | 0.679 [ 0.656, 0.701 ] | 0.676 [ 0.317, 0.742 ] | 0.681 [ 0.656, 0.943 ] |
| 10 | NTDB | 0.571 [ 0.21, 0.662 ] | 0.547 [ 0.499, 0.948 ] | 0.299 [ 0.192, 0.65 ] | 0.92 [ 0.506, 0.961 ] |
| 25 | SweTrau | 0.686 [ 0.621, 0.743 ] | 0.678 [ 0.654, 0.7 ] | 0.684 [ 0.39, 0.742 ] | 0.679 [ 0.655, 0.91 ] |
| 25 | NTDB | 0.595 [ 0.229, 0.668 ] | 0.54 [ 0.501, 0.94 ] | 0.564 [ 0.208, 0.655 ] | 0.556 [ 0.502, 0.955 ] |
| 100 | SweTrau | 0.688 [ 0.627, 0.746 ] | 0.677 [ 0.655, 0.699 ] | 0.686 [ 0.6, 0.745 ] | 0.678 [ 0.655, 0.709 ] |
| 100 | NTDB | 0.606 [ 0.265, 0.676 ] | 0.534 [ 0.497, 0.928 ] | 0.591 [ 0.21, 0.673 ] | 0.541 [ 0.503, 0.952 ] |
| 1000 | NTDB | 0.605 [ 0.28, 0.673 ] | 0.532 [ 0.493, 0.922 ] | 0.594 [ 0.223, 0.672 ] | 0.537 [ 0.496, 0.95 ] |

NTDB, National Trauma Data Bank; SweTrau, Swedish Trauma Registry.

**Table S2. AUC, calibration slope, and calibration intercept**

| **Events per Free Parameter** | **Data set** | **AUC LogReg** | **Calibration Slope LogReg** | **Calibration Intercept LogReg** | **AUC XGBoost** | **Calibration Slope XGBoost** | **Calibration Intercept XGBoost** |
| --- | --- | --- | --- | --- | --- | --- | --- |
| 10 | SweTrau | 0.717 [ 0.676, 0.756 ] | 0.966 [ 0.305, 1.51 ] | -0.379 [ -1.534, 0.329 ] | 0.716 [ 0.632, 0.756 ] | 1.157 [ 0.728, 12.001 ] | -0.134 [ -0.661, 0.753 ] |
| 10 | NTDB | 0.601 [ 0.549, 0.651 ] | 0.96 [ 0.039, 2.59 ] | -0.617 [ -1.352, 0.273 ] | 0.599 [ 0.552, 0.649 ] | 1.288 [ 0.591, 9.412 ] | -0.209 [ -0.668, 0.546 ] |
| 25 | SweTrau | 0.724 [ 0.681, 0.76 ] | 0.977 [ 0.653, 1.267 ] | -0.16 [ -0.742, 0.345 ] | 0.723 [ 0.66, 0.758 ] | 1.062 [ 0.778, 11.532 ] | -0.053 [ -0.522, 0.582 ] |
| 25 | NTDB | 0.61 [ 0.561, 0.655 ] | 0.995 [ 0.278, 1.516 ] | -0.209 [ -1.028, 0.403 ] | 0.605 [ 0.561, 0.651 ] | 1.11 [ 0.643, 8.967 ] | -0.132 [ -0.588, 0.485 ] |
| 100 | SweTrau | 0.726 [ 0.69, 0.758 ] | 1.01 [ 0.837, 1.192 ] | -0.017 [ -0.376, 0.368 ] | 0.725 [ 0.674, 0.759 ] | 1.056 [ 0.839, 8.074 ] | 0.009 [ -0.363, 0.482 ] |
| 100 | NTDB | 0.616 [ 0.568, 0.659 ] | 1.009 [ 0.705, 1.333 ] | -0.038 [ -0.457, 0.414 ] | 0.613 [ 0.569, 0.656 ] | 1.088 [ 0.702, 9.167 ] | -0.063 [ -0.453, 0.426 ] |
| 1000 | NTDB | 0.614 [ 0.572, 0.659 ] | 0.995 [ 0.742, 1.257 ] | -0.015 [ -0.39, 0.384 ] | 0.611 [ 0.572, 0.657 ] | 1.097 [ 0.767, 9.129 ] | -0.021 [ -0.383, 0.391 ] |

NTDB, National Trauma Data Bank; SweTrau, Swedish Trauma Registry.
